# Comparison of different therapeutic approaches for pulmonary cryptococcosis in kidney transplant recipients: a 15-year retrospective analysis

**DOI:** 10.3389/fmed.2023.1107330

**Published:** 2023-07-07

**Authors:** Shuyang Chen, Guoqing Yu, Meiyan Chen, Yanjing You, Lei Gu, Qing Wang, Huijuan Wang, Guoxiang Lai, Zongyang Yu, Wen Wen

**Affiliations:** ^1^Department of Respiratory and Critical Care Medicine, Fuzhou General Hospital of Fujian Medical University, Dongfang Hospital of Xiamen University, The 900th Hospital of the Joint Logistic Support Force, Fuzhou, China; ^2^Department of Pulmonary and Critical Care Medicine, Zhongshan Hospital, Fudan University, Shanghai, China; ^3^Department of Nephrology, Fuzhou General Hospital of Fujian Medical University, Dongfang Hospital of Xiamen University, The 900th Hospital of the Joint Logistic Support Force, Fuzhou, China; ^4^The First Affiliated Hospital of Chongqing Medical University, Chongqing, China; ^5^The Third Affiliated People’s Hospital of Fujian University of Traditional Chinese Medicine, Fuzhou, China

**Keywords:** pulmonary cryptococcosis, post-transplant infection, invasive fungal infection, antifungal therapy, radiographic abnormalities

## Abstract

**Introduction:**

Organ transplant recipients are at increased risk of developing pulmonary cryptococcosis (PC) due to weakened cell-mediated immunity caused by immunosuppressors. However, the nonspecific symptoms associated with PC can often lead to misdiagnosis and inappropriate treatment.

**Methods:**

We conducted a retrospective analysis of data from 23 kidney transplant recipients with PC between April 2006 to January 2021.

**Results:**

The median time from transplantation to the diagnosis of pathology-proven PC 4.09  years. Seventeen patients presented respiratory symptoms, including sputum-producing cough and dyspnea. Additionally, three patients also developed central nervous system (CNS) infections. Chest CT scans frequently revealed nodule-shaped lesions, which can mimic lung carcinoma. Serological tests did not demonstrate any specific changes. Nine patients received surgical resection as treatment. Fourteen patients were treated with antifungal medication only. No recurrence was observed in all 23 patients.

**Conclusion:**

Our study suggests that fever and sputum-producing cough are common symptoms of PC, and cryptococcal meningitis should not be excluded if corresponding symptoms occur. Fluconazole is a common and effective antifungal agent. Surgical resection should be considered for patients who do not respond well to antifungal therapy. Clinicians should be aware of these findings when evaluating transplant recipients with respiratory symptoms.

## 1. Introduction

Opportunistic fungal infections pose a formidable threat to the health and well-being of solid organ transplant (SOT) recipients, particularly due to the use of immunosuppressive therapy and weakened cell-mediated immunity (1). Previous studies have concluded that the incidence rate of cryptococcal infections in SOT was 1%–2% (2–4). Cryptococcal infection is a major burden on the SOT population with the mortality reaching 20% (5).

The Infectious Disease Society of America’s (IDSA) 2010 Clinical Practice Guidelines for the management of cryptococcal infections highlighted 3 populations of concerns: HIV-infected individuals, organ transplant recipients, and HIV-negative/non-transplant-recipients (6). Infected patients often present only nonspecific symptoms or no symptom at all (7). As pulmonary cryptococcosis can exhibit CT presentations similar to lung malignancy, patients often undergo surgical resection and are eventually diagnosed with PC by histopathological examination (8, 9).

After establishing the significance of PC in organ transplant recipients, it is important to further examine the clinical presentation and treatment of PC. A retrospective analysis of clinical data can provide valuable insights into the diagnosis and management of PC in this population.

For this study, we conducted a retrospective study of kidney transplant recipients who developed PC at our institution between 2006 and 2022. Clinical data, including demographic information, immunosuppressive regimen, radiological findings, and laboratory results, were collected and analyzed. Our findings can provide insights into risk factors, clinical presentation, and treatment outcomes of PC in kidney transplant recipients, which can inform clinical decision-making and improve patient outcomes.

## 2. Materials and methods

### 2.1. Patients and diagnostic criteria

From April 2006 to April 2022, kidney transplant recipients with a diagnosis of pulmonary cryptococcosis at hospital discharge were retrospectively reviewed at the 900th Hospital of the Joint Logistic Support Force, Fuzhou, China. Inclusion criteria for this study include: (1) Patients who underwent kidney transplantation at our institution; (2) Patients who were diagnosed with pulmonary cryptococcosis after kidney transplantation; and (3) Patients who had complete clinical data. Exclusion criteria include: (1) Patients who had a history of pulmonary cryptococcosis prior to kidney transplantation; (2) Patients who were diagnosed with other fungal infections; and (3) Patients who had incomplete or missing clinical data. Twenty-three patients were included in the study.

Electronic medical records of all patients were analyzed for demographic data, immunosuppressive regimens, presenting symptoms, therapeutic approaches, follow-up, and outcomes. The diagnosis was established based on the histologic or cytologic identification of *C neoformans* obtained by open biopsy or resection (9 patients), and percutaneous biopsy (12 patients). Two additional patients were diagnosed with cryptococcal infection based on positive cryptococcal antigen test of the cerebrospinal fluid samples.

### 2.2. Image acquisition and evaluation

CT scans were performed with one of the following six CT scanners: Brilliance iCT 256, Philips, US; Brilliance 64, Philips, US; Optima CT660, GE HealthCare, US; LightSpeed VCT, GE HealthCare, US; uCT 510, UnitedImaging, China; uCT S-160, UnitedImaging, China. Scan increments varied from 5 mm to 10 mm. Images were examined with window settings suitable for assessment of the lung parenchyma (Window level, −500 Hounsfield units; window width, 1,500 Hounsfield units).

Chest CT scans were reviewed by at least 2 radiologists for each patient without access to clinical information. Interpretations were reached by consensus. Nodules were defined as lesions with a diameter > 3.0 cm, masses were defined as lesions with a diameter ≤ 3.0 cm. The area of the pulmonary lesion was measured using the Picture Archiving and Communication System (PACS) (The 900th Hospital, INFINITT SH Co., Ltd.). For a single lesion, two perpendicular diameters on the plane with the largest lesion was measured. For multiple lesions, the largest cross-section of the largest lesion was measured. Lesion characteristics and associated findings were also evaluated and recorded. CT scans were assessed for ground-glass opacity, halo sign, pleural indentation, etc.

**Figure 1:**
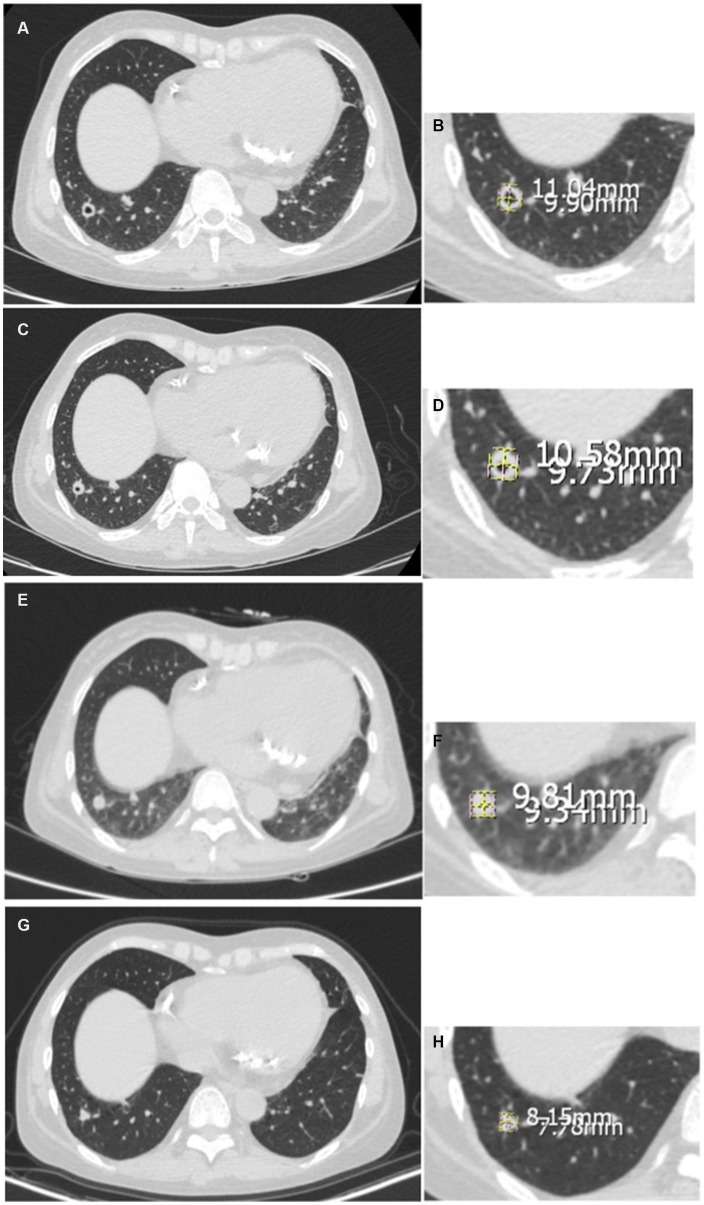
Measurement of the pulmonary lesion diameter. **(A–H)** The case of a 45-year-old female patient diagnosed with PC, indicating the changes over a 12-month follow-up evaluation (onset, 6 months, 9 months, 12 months, respectively).

Follow-up CT scans were performed in 23 patients at 3, 6, 9, 12 months after the initiation of antifungal therapy or the surgery. Lesions were measured with the same method stated as before. Measurements from the largest cross-section of the lesion were used in statistical analysis (Figure 1). For multiple lesions, we make sure to measure the same lesion in the same location. As measuring the size of lesions in different CT may not be absolutely accurate, radiologists were also asked to evaluate the lesions as Complete Resolution (disappearance of radiographic evidence of pulmonary abnormalities), Deterioration (progressive radiographic worsening of pulmonary abnormalities), Partial Regression (decrease in the size of the radiographic presentation of pulmonary abnormalities), or Stable (no clear change when compared with previous scans).

### 2.3. Statistical analysis

Continuous variables, including time-related data, laboratory counts, and changes in CT images are reported with mean values and standard deviations and were compared using Student’s t test. Categorical variables are reported as numbers and proportions and were compared between groups employing either chi-squared test or Fisher exact test. *p* values < 0.05 was considered as a statistically significant difference (two-tailed).

All data were analyzed using SPSS 26.0 (SPSS Inc., Chicago, IL, United States).

### 2.4. Patient and public involvement statement

This study is a retrospective analysis, patients gave informed consent for the use of their medical data. This article contains no personal or(and) private information of the patients involved.

This study was approved by the Ethics Committee of the 900th Hospital of Joint Logistic Forces (Approval Number: 20200201CR).

## 3. Results

### 3.1. Demographics and clinical data

Patient demographic and clinical data are summarized in Table 1. 18 patients were male and 5 were female. The average time between kidney transplant and cryptococcal infection was 1433.05 days (3.92 years). In terms of underlying kidney diseases, the most common one was Hypertension (9, 39.1%).

**Table 1:** Demographics and clinical information of patients with pulmonary cryptococcosis* (*n* = 23).

Variables	Patients (*n* = 23)
**Gender**	
Male	18 (78.3)
Female	5 (21.7)
**Key time indicators**	
Age of kidney transplantation^a^	41.41 ± 13.40
Age of PC diagnosis^a^	45.40 ± 12.79
Time between transplant to infection^b^	1433.05 ± 1038.75 (3.92 ± 2.84 years)
**Underlying kidney diseases**	
Hypertension	9 (39.1)
Type 2 diabetes mellitus	5 (21.7)
**Immunosuppressive regimen**	
Calcineurin inhibitor (CNI)	20 (87. 0)
Tacrolimus	15 (65.2)
Cyclosporin A	5 (21.7)
Sirolimus	3 (13.0)
Glucocorticoid	19 (82.6)
**Type of disease**	
Confined (pulmonary)	20 (86.9)
Dissemination	3 (13.1)
**Selected presenting symptoms**	
Asymptomatic	6 (26.1)
Productive cough	11 (47.8)
Shortness of breath	2 (8.7)
Pharyngalgia	3 (13.0)
Fever	4 (17.4)
Headache	2 (8.7)
Vomiting	1 (4.3)
**Therapeutic approaches**	
Antifungal drugs	14 (60.9)
Surgery	4 (17.4)
Antifungal drugs + surgery	5 (21.7)

*Values are given as no. of patients (%), unless otherwise indicated.

a Values are given as the mean ± SD, years old.

b Values are given as the mean ± SD, days.

All patients routinely accepted immunosuppressive treatments following kidney transplantation, regimens used for these patients are listed in Table 1. Calcineurin inhibitor (CNI) was maintained in 20 patients, 3 patients received Sirolimus for CNI sparing. Glucocorticoid was maintained in 19 patients. None were HIV-positive.

Productive cough was the most common presenting symptom, occurring in 11 patients, followed by fever (17.4%), pharyngalgia (13%) and shortness of breath (8.7). 6 patients were asymptomatic. Cryptococcal infection was confined to the lungs in 21 patients, whereas dissemination occurred in 3 patients, 2 in CNS (cryptococcal meningitis) and 1 in skin (cutaneous cryptococcosis).

14 patients were treated conservatively. 4 patients received surgical therapy. 5 patients received unsatisfactory antifungal therapy prior to surgical intervention. None of the patients died of cryptococcosis. None of the patients reported possible exposure to pigeon droppings.

### 3.2. Initial chest CT features

**Figure 2:**
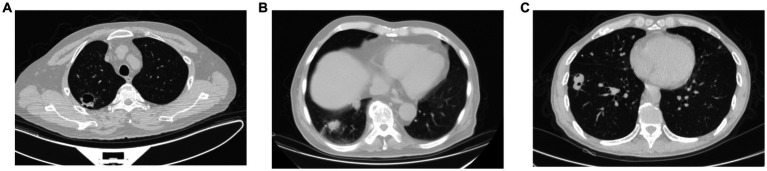
Chest CT in the lung window showing different pulmonary radiographic abnormalities. **(A)** Spiculation presented around pulmonary lesion. **(B)** A cavity formed within the nodule-shaped lesion. **(C)** Halo sign presented around the pulmonary lesion.

Radiographic pulmonary abnormalities observed on the initial CT scans were summarized in Table 2. The most common finding was pulmonary mass/nodule. Multiple lesions were observed in only 1 patient. Most nodule-shaped lesions had poorly-defined margins. These pulmonary lesions predominately locate in the middle lobe of the right lung. The presence of spiculation can be observed in 14 patients (60.9%, Figure 2A), followed by cavitation (34.8%, Figure 2B) and halo sign (8.7%, Figure 2C). One patient also developed pleural effusion. No ground-glass opacity or mediastinal lymphadenopathy was observed.

**Table 2:** Image presentations and characteristics* (*n* = 23).

Findings	Patients (*n* = 23)
Pulmonary masses	8 (34.8)
Pulmonary nodules	15 (65.2)
Solitary	14 (60.9)
Multiple	1 (4.3)
with spiculation	14 (60.9)
with cavitation	8 (34.8)
with halo sign	2 (8.7)
**Associated findings**	
Pleural effusion	2 (8.7)
**Lobes infected**	
Right, Superior Lobe	4 (17.4)
Right, Middle Lobe	10 (43.3)
Right, Inferior Lobe	3 (13.0)
Left, Superior Lobe	4 (17.4)
Left, Inferior Lobe	2 (8.7)

*Values are given as no. of patients (%), unless otherwise indicated.

### 3.3. Treatment, outcomes and follow-up studies

Patients were divided into 3 groups based on different therapeutic approaches: **Antifungal** group, **Surgical** group, and **Antifungal + Surgical** group. Table 3 summarizes the therapeutic approaches and radiographic surveillance studies conducted during the one-year follow-up period for all patients.

**Table 3:** Summary of therapeutic approaches and radiographic surveillance studies during one-year follow-up* (*n* = 23).

Patients	Initial CT Scan Findings	Treatment	3-mo CT F/U			6-mo CT F/U			9-mo CT F/U			12-mo CT F/U			Outcome
			Imp	Sta	Det	Imp	Sta	Det	Imp	Sta	Det	Imp	Sta	Det	
**Antifungal group**															
1	SN	Azole, 10 mo	+			NA			+			+			CR
2	SN	Azole, 5 mo		+			+		NA			NA			Stable
3	SC	Azole, 3 mo		+			+				+			+	Deterioration
4	SC	Azole, 5 mo	+			+			+			+			PR
5	SN	Azole, 6 mo	+			+			NA			+			CR
6	MN	Azole, 11 mo		+			+		NA				+		Stable
7	SN	Azole, 3 mo	+			+			+			NA			PR
8	SN	IV ampho	+				+			+		+			PR
9	SN	Azole, 3 mo	+			+			+			+			PR
10	SN	Azole, 5 mo			+		+			+			+		Deterioration
11	SN	Azole, 2 mo	+			+			+			+			PR
12	SC	Azole, 12 mo		+				+		+			+		Deterioration
13	SC	Azole, 12 mo	+			+			+			+			CR
14	SN	Azole, 5 mo	+			+			+			+			PR
**Surgical group**															
15	SN	Surgery + IV vori	NA			+			NA			NA			CR
16	SC	Surgery + IV vori	NA			NA			+			NA			CR
17	SN	Surgery	NA			+			NA			NA			CR
18	SN	Surgery+Azole, 2 mo	NA			NA			NA			+			CR
**Antifungal + surgical group**															
19	SC	Azole, 12 mo + Surgery	+			NA			NA			+			CR
20	SN	Azole, 1 mo + Surgery	NA			+			+			NA			CR
21	SC	IV ampho + Surgery	NA			+			+			NA			CR
22	SC	Azole, 8 mo + Surgery	+			NA			NA			+			CR
23	SN	Azole, 1 mo + Surgery	NA			+			+			NA			CR

*ampho, amphotericin; Azole, oral fluconazole; CR: complete regression; Imp, improved; Sta, stable; Det, deterioration; vori, oral Voriconazole; MN, multiple nodules; SC, solitary consolidation; SN, solitary nodule; mo, month; NA, Not Applicable; +, condition present.

13 patients in the **Antifungal** group received fluconazole as the initial treatment, with 2 patients receiving a daily dosage of 200 mg, and the remaining 11 patients receiving a daily dosage of 400–600 mg. 1 patient received intravenous amphotericin B as the initial treatment due to disseminated cryptococcal infection. The median duration of antifungal therapy was 5 months (range: 2–12 months).

All Patients in the **Surgical** group received lobectomy. 3 patients also received post-operation antifungal therapy, including fluconazole and voriconazole. 1 patient received no additional antifungal therapy. No patient in the surgical group exhibited recurrence during the one-year follow-up study.

**Figure 3:**
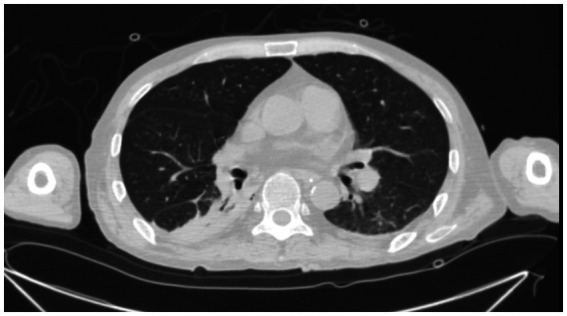
A 45-year-old male PC patient with a history of kidney transplantation 3 years ago. Chest CT in the lung window shows pleural effusion 3  days after lobectomy.

The **Antifungal + Surgical** group consists of patients who received unsatisfactory initial antifungal therapy prior to a surgical procedure. 4 patients received fluconazole prior to surgical intervention. The duration of antifungal therapy in this group varied from 1 month to 12 months (median duration: 8 months). 2 patients who received lobectomy later developed pleural effusion (Figure 3) and post-operation infections.

Blood tests were not able to determine abnormal changes in this cohort of patients. 5 patients were tested for serum cryptococcal capsular antigen (CrAg) and yielded positive results. In 2 cases with CNS cryptococcosis infections, cryptococcal capsular antigen(CrAg) was positive in cerebrospinal fluid samples.

Patients were asked to make follow-up visits at 3 months, 6 months, 9 months, and 12 months after the initiation of antifungal therapy or the surgery. Chest CT was performed at each follow-up visit. Of the patients in the **Antifungal** group at their 3-month follow-up evaluation, 9 patients showed improvement of pulmonary abnormalities, the conditions of 4 patients remained unchanged, and 1 patient showed radiographic deterioration. At the 6-month follow-up evaluation, 7 patients showed radiographic improvement while 5 patients remained radiographically stable, and 1 patient showed deterioration. At the 12-month evaluation, 8 patients showed significant resolution of pulmonary abnormalities, while 1 patient showed radiographic deterioration. In the **Surgical** and the **Antifungal** + **Surgical** groups, no patient had radiographic recurrence after 12 months of follow-up.

## 4. Discussion

Our study intended to discover and compare the different outcomes of PC patients with different therapeutic approaches. Consistent with most of the researches on PC, cryptococcal infection following a kidney transplant is a late-occurring disease, with most of our patients infected 2  years after transplantation (10). In a retrospective analysis that included patients over the past 30 years, the occurring time was 5.16 ± 3.97 years (11). The occurring time in our study was 3.92 ± 2.84 years, which was in agreement with previous studies. Different occurring time may indicate different origins of the infection. A latent infection may be reactivated and lead to a late occurred infection, while an early infection may indicate donor-sourced infection. Donor-derived infection should be considered a possibility when patients showing symptoms within the first few months after transplantation (12).

The most common underlying kidney disease in this group of patients is hypertension, while type 2 diabetes mellitus is also quite common. Previous large-scale retrospective analysis also reported similar patterns of underlying kidney diseases (4). Due to the immunosuppressors, kidney transplant recipients are a group of patients with relatively higher risks of developing diabetes mellitus (DM), which is an independent factor contributing to the morbidity and mortality of cryptococcal infections (13). A study in China concluded kidney transplant recipients had an elevated risk for CNS cryptococcal infection (14). CNS-related cryptococcal infection can be far more severe, cases reported elsewhere include patients presenting symptoms like seizures, altered mental status, and can be fatal despite aggressive treatment. Although none of the patients included in this cohort reported exposure to pigeon droppings or residing in close proximity to pigeon habitats, it is imperative to acknowledge that cryptococcal infections often originate from direct or circumstantial contact with pigeon guano (15). Hence, it assumes paramount importance to educate immunocompromised patients about the criticality of abstaining from activities that might potentially expose them to such sources of infection. Furthermore, adopting suitable protective measures becomes integral in safeguarding their well-being.

In the care of renal transplant patients, it’s extremely important for transplant recipients and healthcare providers to maintain a high level of suspicion for even nonspecific and ambiguous symptoms. Our study found that the most commonly observed symptoms in PC patients were productive cough and fever, which lack specificity in relation to invasive fungal diseases. Nonetheless, it is noteworthy that 2 patients, afflicted with CNS cryptococcal infection, experienced a more severe clinical course characterized by elevated fever, headache, and vomiting. Interestingly, 6 patients were asymptomatic and were found incidentally during chest CT examinations. These observations underscore the complexities inherent in diagnosing invasive fungal complications within the immunocompromised hosts, particularly among kidney transplant recipients who receive ongoing immunosuppressive therapy. The study’s findings also suggest the potential value of regular CT scans in immunocompromised patients. However, it is imperative to acknowledge the current absence of definitive recommendations regarding appropriate follow-up protocols, including imaging examinations, for kidney transplant recipients at an elevated risk of encountering invasive fungal infections. Consequently, substantial variation exists within clinical practices, with decisions concerning follow-up procedures relying heavily upon the individual discretion of healthcare providers and the protocols established within their respective institutions.

**Figure 4:**
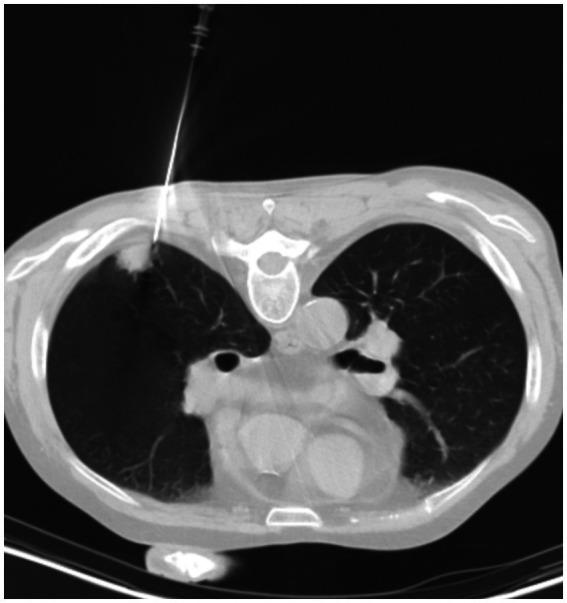
A 58-year-old male PC patient with a history of kidney transplantation 2 years ago. Chest CT in the lung window shows the process of percutaneous lung needle biopsy (PCNB).

Considering most PC patients only present non-specific symptoms or no symptom at all, diagnosis of PC is often delayed or even mistakenly diagnosed and/or treated as lung carcinoma. While radiographic findings are important in understanding the nature and extent of the lesions, convincing diagnosis of PC relies on histopathology, culture-based methods and cryptococcal antigen (CrAg) testing (15). The diversified radiographic presentation of PC patients may be confusing. It’s also common for pulmonary radiographic abnormalities to be provisionally diagnosed as pulmonary malignancy (15), such as patients in the **Surgical** group of our study. 12 patients in the **Antifungal** group were diagnosed using CT-guided percutaneous lung needle biopsy (PCNB, Figure 4), which facilitates accurate diagnosis and exclusion of differentials. In addition to invasive diagnostic technique, serum CrAg test in non-human immunodeficiency virus adult PC patients has been found to be useful, especially in patients with extensive lung involvement (16). However, study also indicated that patients’ immune status and radiography would affect the detection of cryptococcal antigen (17). Nevertheless, CrAg has extremely high sensitivity and specificity in cerebrospinal fluid (CSF) (18). Guidelines suggested that sampling of CSF is a must to exclude CNS infection in all PC patients (6, 19). Metagenomic next-generation sequencing (mNGS) is a potentially new diagnostic tool for PC patients. A recent study suggested that while the sensitivity of mNGS is lower than that of serum CrAg and histopathology in immunocompetent patients, bronchoalveolar lavage fluid (BALF) mNGS detection is recommended for immunocompromised patients (20).

The choice of therapeutic approaches is dependent on multiple factors like primarily immune status and the severity of the disease (6, 21). In the guideline published by the Infectious Disease Society of America (IDSA) in 2010, the surgical approach was only recommended for patients showing persistent radiographic abnormalities and symptoms nonresponding to antifungal therapy (6). Another possibility for the abundant use of surgical removal as a therapeutic approach in this cohort is that diagnosis of lung carcinoma was made based on radiographic abnormalities. A study involving 53 patients concluded that surgical resection was required in some immunocompetent patients to diagnose and effectively treat PC (8). Although no surgically-treated patients in this study experienced recurrence, we still believe surgical therapy is not the optimal therapeutic selection for PC especially for transplant recipients. Among 9 surgically-treated patients, only 3 of them received postoperative antifungal treatment, while current study suggests a 2-month postoperative antifungal treatment to facilitate an optimal outcome (22).

Management of cryptococcal infection in transplant recipients is of a special challenge as drug–drug interactions may occur. For example, when CNI are co-administered with fluconazole, therapeutic drug monitoring should be performed due to known drug–drug interaction (23). Current guidelines suggest the use of oral fluconazole at a dosage of 400 mg/day for 6–12 months (6). Only 4 patients in the **Antifungal** group followed through the recommended therapy. Deterioration of pulmonary radiographic abnormalities may be associated with the shortened antifungal treatment duration except for patient 12. A real-world study involving patients with different immune statuses indicated that following the recommended treatment regimen can reduce mortality in PC patients (24). IDSA guidelines also suggest a sequential or step-wise reduction of immunosuppressants, suggesting the dose of the corticosteroid to be lowered first. While further clinical studies should be performed to validate the effectiveness of these suggestions, it is of great necessity to determine whether immunosuppressants influence the pharmaceutical effects of antifungal therapy (6).

Our study has several limitations. First, this work was retrospective in nature and was performed in a single medical center with limited cases. The cohort in the study was included completely based on their discharge diagnosis, which was ascertained based on serological and pathological identification. However, it’s of paramount importance to recognize the possible selection bias inherent in this approach. Nonetheless, as our study mainly concentrated on evaluation and comparison of different therapeutic approaches, we maintain the belief that our work engenders convincing results, even in light of the potential presence of such bias. Second, the evaluation of radiographic changes in each follow-up visit was obtained by manually measuring the lesion. This may be inaccurate even when the measurement was averaged by separate radiologists. Third, CrAg tests and titers were scarcely used for our patients, dynamically monitoring the changes of CrAg titers may indicate the burden of PC.

Pulmonary cryptococcosis is an important opportunistic fungal infection among SOT recipients. PC varies in symptoms and lacks specific clinical presentations, requiring physicians to be particularly vigilant when immunocompromised or immunosuppressed patients develop respiratory disease-related symptoms. Evidence-based research continuously provides new insights into the treatment and management of these patients. Future studies should focus on the specific and targeted diagnostic method for potential PC cases in SOT recipients.

## Data availability statement

The non-identifiable data from the 900^th^ Hospital that support the findings of this study are available upon reasonable request from the corresponding author (WW).

## Ethics statement

The studies involving human participants were reviewed and approved by Ethics Committee of the 900th Hospital of Joint Logistic Forces (Approval Number: 20200201CR). The patients/participants provided their written informed consent to participate in this study.

## Author contributions

SC, GY, and WW: conceptualization. SC, LG, and QW: methodology. SC and MC: investigation. SC, GY, YY, HW, GL, and ZY: formal analysis. SC, GY, and MC: writing – original draft. WW, SC, GY, and MC: writing – review & editing. WW: funding acquisition. GL and ZY: supervision. All authors contributed to the article and approved the submitted version.

## Funding

This research was supported by Fujian Scientific Innovation Joint Funding (2020Y9042 to WW).

## Conflict of interest

The authors declare that the research was conducted in the absence of any commercial or financial relationships that could be construed as a potential conflict of interest.

## Publisher’s note

All claims expressed in this article are solely those of the authors and do not necessarily represent those of their affiliated organizations, or those of the publisher, the editors and the reviewers. Any product that may be evaluated in this article, or claim that may be made by its manufacturer, is not guaranteed or endorsed by the publisher.
